# A hybrid deep CNN model for brain tumor image multi-classification

**DOI:** 10.1186/s12880-024-01195-7

**Published:** 2024-01-19

**Authors:** Saravanan Srinivasan, Divya Francis, Sandeep Kumar Mathivanan, Hariharan Rajadurai, Basu Dev Shivahare, Mohd Asif Shah

**Affiliations:** 1 Department of Computer Science and Engineering, Vel Tech Rangarajan Dr.Sagunthala R&D Institute of Science and Technology, Chennai, 600062 India; 2 Department of Electronics and Communication Engineering, PSNA College of Engineering and Technology, Dindigul, 624622 India; 3 School of Computing Science and Engineering, Galgotias University, Greater Noida, 203201 India; 4 School of Computing Science and Engineering, VIT Bhopal University, Bhopal–Indore Highway Kothrikalan, Sehore, 466114 India; 5 Department of Economics, Kabridahar University, Po Box 250, Kebri Dehar, Ethiopia; 6 Centre of Research Impact and Outcome, Chitkara University Institute of Engineering and Technology, Chitkara University, Rajpura, Punjab 140401, India; 7 Division of Research and Development, Lovely Professional University, Phagwara, Punjab 144001, India

**Keywords:** Brain tumor grading, Hybrid deep learning, Hybrid convolutional neural network, Grid search, Hyperparameters

## Abstract

The current approach to diagnosing and classifying brain tumors relies on the histological evaluation of biopsy samples, which is invasive, time-consuming, and susceptible to manual errors. These limitations underscore the pressing need for a fully automated, deep-learning-based multi-classification system for brain malignancies. This article aims to leverage a deep convolutional neural network (CNN) to enhance early detection and presents three distinct CNN models designed for different types of classification tasks. The first CNN model achieves an impressive detection accuracy of 99.53% for brain tumors. The second CNN model, with an accuracy of 93.81%, proficiently categorizes brain tumors into five distinct types: normal, glioma, meningioma, pituitary, and metastatic. Furthermore, the third CNN model demonstrates an accuracy of 98.56% in accurately classifying brain tumors into their different grades. To ensure optimal performance, a grid search optimization approach is employed to automatically fine-tune all the relevant hyperparameters of the CNN models. The utilization of large, publicly accessible clinical datasets results in robust and reliable classification outcomes. This article conducts a comprehensive comparison of the proposed models against classical models, such as AlexNet, DenseNet121, ResNet-101, VGG-19, and GoogleNet, reaffirming the superiority of the deep CNN-based approach in advancing the field of brain tumor classification and early detection.

## Introduction

Brain tumors stand as one of the leading causes of death in the modern world. These tumors can manifest in various regions of the brain, often remaining asymptomatic until later stages of life. Symptoms of brain disease encompass a wide array of issues, including personality changes, memory difficulties, communication impairments, hearing or speech challenges, chronic migraines, and even vision loss [1]. Notable examples of brain tumors include meningiomas, gliomas, pituitary adenomas, and acoustic neuromas. According to medical observations, meningiomas, gliomas, and pituitary tumors account for approximately 15%, 45%, and 15% of all brain tumors, respectively. A brain tumor can have long-lasting psychological effects on the patient. These tumors originate from primary abnormalities in the brain or central spine tissue that disrupt normal brain function. Brain tumors are classified into two main categories: benign and malignant. Benign tumors grow slowly and are non-cancerous; they are relatively rare and do not metastasize. In contrast, malignant brain tumors contain cancerous cells, typically originating in one region of the brain before swiftly spreading to other areas of the brain and spinal cord [2]. Malignant tumors pose a significant health risk. The World Health Organization (WHO) classifies brain tumors into four grades based on their behavior within the brain: grades 1 and 2 are considered low-grade or benign tumors, while grades 3 and 4 are categorized as high-grade or malignant tumors. Several diagnostic methods, such as CT scanning and EEG, are available for detecting brain tumors, but magnetic resonance imaging (MRI) is the most reliable and widely utilized. MRI generates detailed internal images of the body’s organs by employing strong magnetic fields and radio waves [3]. Essentially, CT or MRI scans can distinguish the affected brain region due to the tumor from the healthy tissue. Biopsies, clinical tests that extract brain cells, can be conducted as a prelude to cerebral surgery. Precision is paramount in measuring tumor cells or arriving at accurate diagnoses. The emergence of machine learning (ML) presents an opportunity to assist radiologists in furnishing precise disease status information [4]. The proliferation of novel technologies, particularly artificial intelligence and ML, has left an indelible mark on the medical field, equipping various medical departments, including medical imaging, with indispensable tools to enhance their operations. As MRI images are processed to aid radiologists in decision making, a diverse array of automated learning strategies is employed for classification and segmentation purposes. While supervised methods for classifying brain tumors hold immense promise, they demand specialized expertise to optimize the feature extraction and selection techniques [5]. In navigating and analyzing vast datasets, expert medical professionals benefit from the support of machine assistance. Furthermore, the failure to accurately identify life-threatening tumors could potentially result in treatment delays for patients. The utilization of deep-learning (DL) techniques in detecting brain tumors and extracting meaningful insights from data patterns has a longstanding history. DL’s capability to classify and model brain cancers is widely recognized [6]. Effectively treating brain tumors hinges on early and precise disease diagnosis. Decisions regarding treatment methods are influenced by factors such as the tumor’s pathological type, grade, and stage at diagnosis. Neuro-oncologists have harnessed computer-aided diagnostic (CAD) tools for various purposes, including tumor detection, categorization, and grading within the realm of neurology [7].

A glioma is a type of tumor that originates in brain tissue, distinct from nerve cells or blood vessels. In contrast, meningiomas develop from the protective membranes that envelop the brain and central nervous system, while pituitary tumors grow within the confines of the skull. Among these three tumor types, meningiomas are relatively rare and generally benign. Conversely, gliomas constitute the most prevalent form of malignant brain tumors. Even though pituitary tumors may be benign, they can still give rise to significant medical complications [8]. Brain tumors rank as a leading cause of mortality worldwide. Research underscores the significance of early and accurate identification, coupled with prompt treatment, in improving survival rates for patients with cancerous tumors. In certain instances, healthcare professionals may encounter the need to differentiate between strokes and tumors. Hence, the early detection of brain tumors assumes pivotal importance for providing effective care and potentially extending the affected individual’s lifespan [9]. Convolutional neural networks (CNNs), distinguished by their multi-layered architecture and high diagnostic accuracy when provided with ample input images, currently stand as a highly effective approach in image processing. Neural networks, including auto-encoders, an unsupervised learning technique, are harnessed for representation learning [10]. Magnetic resonance imaging (MRI) emerges as an exceptional tool for obtaining clear and detailed visualizations within the human body. Unlike X-rays or CT scans that involve ionizing radiation, MRI offers significantly enhanced contrast between various soft tissues. Moreover, MRI technology furnishes detailed images from multiple angles, providing radiologists with abundant data on human soft-tissue anatomy [11]. The aim of this paper is to introduce three fully automatic CNN models designed for the multi-classification of brain tumors, utilizing publicly available datasets. To the best of the authors’ knowledge, this represents the first endeavor in multi-classifying brain tumors from MRI images using CNNs, wherein nearly all the hyperparameters are automatically tuned through the grid search optimizer. The rest of this paper is organized as follows: Introduction Section: this section provides a comprehensive overview of various tumor types and their diagnostic methods; Related work Section: in this section, we delve into recent articles, examining their methods, outcomes, and applications; Materials and methods Section: here, we detail the utilization of datasets and describe the proposed model architectures; Experimental study Section: this section centers on a comparative analysis of the accuracies achieved by our proposed method and other state-of-the-art approaches;  Conclusions Section: this section offers the concluding remarks and insights related to our proposed model.

## Related work

The author’s goal was to devise a classification approach that is notably more accurate, cost-effective, and self-training, utilizing an extensive collection of authentic datasets rather than augmented data. The customized VGG-16 (Visual Geometry Group) architecture was employed to classify 10,153 MRI images into three distinct classes (glioma, meningioma, and pituitary). The network demonstrated a remarkable performance, achieving an overall accuracy of 99.5% and precision rates of 99.4% for gliomas, 96.7% for meningiomas, and 100% for pituitaries [12]. The proposed model’s efficacy was assessed using three CNN models: AlexNet, Visual Geometry Group (VGG)-16, and VGG-19. AlexNet achieved a peak detection accuracy of 99.55% using 349 images sourced from the Reference Image Database to Evaluate Response (RIDER) neuro MRI database. For brain tumor localization, employing 804 3D MRIs from the Brain Tumor Segmentation (BraTS) 2013 database, a Dice score of 0.87 was achieved [13]. In the investigation of brain tumor categorization, an array of deep- and machine-learning techniques, including softmax, Random Forest, Support Vector Machine (SVM), K-Nearest Neighbors, and the ensemble method, were employed. These outcomes were compared with existing methods. Notably, the Inception-v3 model exhibited the highest performance, attaining a test accuracy of 94.34%. This advancement holds the potential to establish a prominent role in clinical applications for brain tumor analysis [14]. An effective approach was proposed for categorizing brain MRIs into four classes: normal and three forms of malignant brain tumors (glioblastoma, sarcoma, and metastatic bronchogenic carcinoma). The method integrates the discrete wavelet transform (DWT) with a deep neural network (DNN). Employing a deep neural network classifier, one of the DL designs, a dataset of 66 brain MRIs was classified into the specified categories. The integration of DWT, a powerful feature extraction technique, principal component analysis (PCA), and the classifier yielded commendable performances across all evaluation metrics [15]. The author introduced a strategy involving a CNN to distinguish brain tumors from 2D MRI scans of the brain. This initial separation is subsequently followed by the application of conventional classifiers and DL techniques. In addition, an SVM classifier, along with various activation algorithms, such as softmax, RMSProp, and sigmoid, were employed to validate and cross-check the proposed approach. The implementation of the author’s suggested solution was executed using TensorFlow and Keras in the Python programming language, chosen for its robust capabilities in expediting tasks. The achieved accuracy rate for the CNN model stood at an impressive 99.74% [16]. This paper presents a brain tumor classification approach employing open-access datasets and CNN techniques. The methodology utilizes open-access datasets to classify tissue as either tumor or non-tumor through a distinctive framework that combines discrete cosine transform-based image fusion, CNN super-resolution, and a classifier. Employing super-resolution and the ResNet50 architecture, the framework attained an impressive accuracy of 98.14% [17].

A novel approach for dimensionality reduction is proposed, utilizing the Grey Wolf Optimizer (GWO) and rough-set theory. This method identifies relevant features from extracted images, distinguishing between high-grade (HG) and low-grade (LG) glioblastoma multiforme (GBM) while accommodating feature correlation constraints to eliminate redundant attributes. Additionally, the article introduces a dynamic architecture for multilevel layer modeling in a Faster R-CNN (MLL-CNN) approach. This is achieved using a feature weight factor and a relative description model to construct selected features, thereby streamlining the processing and classifying of long-tailed files. This advancement leads to improved training accuracies for CNNs. The findings illustrate that the overall survival prediction for GBM brain growth achieves a higher accuracy of 95% and a lower error rate of 2.3% [18]. The work involves the classification of 253 high-resolution brain MR images into normal and pathological classes. To efficiently and accurately train deep neural models, MR images were scaled, cropped, pre-processed, and enhanced. The Lu-Net model is compared against LeNet and VGG-16 using five statistical metrics: precision, recall, specificity, F-score, and accuracy. The CNN models were trained on enhanced images and validated on 50 sets of untrained data. LeNet, VGG-16, and the proposed approach achieved accuracy rates of 88%, 90%, and 98%, respectively [19]. MIDNet18 outperformed AlexNet in categorizing brain tumor medical images. The proposed MIDNet18 model demonstrated effective learning, achieving a binary classification accuracy exceeding 98%, which is statistically significant (independent-sample *t*-test, *p* < 0.05). MIDNet18 excelled across all the performance indicators for the dataset used in this study [20].

The objective of this study was to facilitate accurate early-stage diagnoses by medical professionals. Three DL architectures—AlexNet, GoogLeNet, and ResNet50—were employed to identify brain tumor images. Among them, the ResNet50 architecture demonstrated the highest accuracy rates. The experimental results yielded an accuracy of 85.71%, with the potential for further enhancement in future research [21]. In the realm of Alzheimer’s disease diagnosis, the CNN approach was utilized to detect patients using MRSI and supplementary MRI data. High Matthews Correlation Coefficient (MCC) scores were achieved, with area-under-the-curve values of 0.87 and 0.91 for MRSI and MRI, respectively. A comparative analysis highlighted the superiority of Partial Least Squares and Support Vector Machines. The proposed system automatically selected critical spectral regions for diagnosis, corroborating findings with literature biomarkers [22]. CNNs, ML pipelines inspired by biological neural processes, have been extensively studied. The author’s approach involved first acquiring an understanding of CNNs, followed by a literature search for a segmentation pipeline applicable to brain tumor segmentation. Additionally, the potential future role of CNNs in radiology was explored. The application of CNNs was demonstrated in predicting survival and medication responses through analyses of the brain tumor shape, texture, and signal intensity [23]. In this paper, the state-of-the-art object detection framework YOLO (You Only Look Once) was employed to identify and classify brain tumors using DL. YOLOv5, a revolutionary object detection algorithm, stood out for its computational efficiency. The RSNA-MICCAI brain tumor radiogenomics classification BraTS 2021 dataset served as the basis. YOLOv5 achieved an 88% precision rate [24]. The primary aim of this method is to classify brain images as healthy or tumorous using test MRI data. MRI-based brain tumor research offers superior internal imaging compared to CT scans. The approach involves denoising MRI images with an anisotropic diffusion filter, segmenting using morphological operations, and classifying via a five-layer CNN-based hybrid technique, outperforming other methods. The developed model, utilizing the publicly available KAGGLE brain MRI database, achieved an accuracy rate of 88.1% [25]. The adoption of AI-powered computer systems can assist doctors in making more accurate diagnoses. In this research, we developed a brain tumor diagnostic system based on CNN technology, utilizing Ranger optimization and the extensive pre-processing of data from the EfficientNetv2 architecture [26]. This research introduces a novel topology for a parallel deep CNN (PDCNN) designed to extract both global and local features from two parallel stages. Overfitting is addressed through the utilization of dropout regularization and batch normalization. Unlike conventional CNNs that collect features randomly without considering local and global contexts, our proposed PDCNN architecture aims to capture a comprehensive range of features [27]. This study focuses on the classification of meningiomas, gliomas, and pituitary tumors using MRI imaging. The Dual VGG-16 CNN, equipped with a proprietary CNN architecture, constitutes the DCTN mode [28]. The importance of the early detection of brain tumors cannot be overstated. Biopsies of brain tumors, the gold standard for diagnosis, are only possible during life-altering brain surgery. Methods based on computational intelligence can aid in the diagnosis and categorization of brain tumors [29]. The author employed a DL model to classify MRI scans into glioma and normal categories, preceded by the extraction of scan information. Convolutional recurrent neural networks (CRNNs) were utilized for generating the classifications. This suggested method significantly improved the categorization of brain images within a specified input dataset [30]. The network was trained and tested using BraTS2019 data. The approach was evaluated using the Dice similarity coefficient (DSC), sensitivity (Sen), specificity (Spec), and Hausdorff distance (HD). The DSCs for the entire tumor, tumor core, and enhancing tumor were 0.934, 0.911, and 0.851, respectively. The subregion Sen values were 0.922, 0.911, and 0.867. The Spec and HD scores were 1.000, 1.000, and 3.224, 2.990, 2.844, respectively [31]. The cancer region segmentation from brain images is achieved using Deep K-Net, a hybrid approach that combines K-Net and utilizes Deep Joint Segmentation with Ruzicka similarity. The K-Net is trained using a Driving Training Taylor (DTT) algorithm. The DTT algorithm optimizes the Shepard CNN (ShCNN) for classification [32].

The author provided an overview of contemporary computer-aided detection methods that utilize WCE images as input, distinguishing them as either diseased/abnormal or disease-free/normal. We conducted an evaluation of approaches designed for the detection of tumors, polyps, and ulcers, as these three conditions are categorized similarly. Furthermore, because general abnormalities and bleeding within the GI tract could be indicative of these disorders, we made an effort to shed light on the research conducted for the identification of abnormalities and bleeding within WCE images [33]. Author have included several research studies, each accompanied by detailed descriptions of their techniques, findings, and conclusions. Additionally, we provide a discussion and comparison of previous review articles, which serves as a reference point for the current survey, while also highlighting its limitations [34]. To enhance feature extraction, our proposed deep CNN model introduces an innovative approach by incorporating multiple convolutional kernels with varying window widths within the same hidden layer. This architecture is designed to be lightweight, consisting of 16 convolutional layers, 2 fully connected layers (FCN), and a softmax layer serving as the output layer. The activation function employed in the first 15 layers is MISH, followed by the Rectified Linear Unit (ReLU) activation function. This combination not only facilitates profound information propagation but also offers self-regularized, smoothly non-monotonic characteristics, while effectively mitigating saturation issues during training. The authors present a comprehensive set of experimental results, comparing our model’s performance against benchmarks like the MICCAI 2015 challenge and other publicly available datasets. Our findings demonstrate that the proposed model excels in terms of accuracy, sensitivity, the F1-score, the F2-score, and the Dice coefficient [35].

## Materials and methods

### Materials

The study used four different datasets that can be found in freely accessible databases. The Figshare dataset is the name of the first dataset. From 19 patients with glioblastomas (G-IV), MRI multi-sequence images were taken and added to the Figshare dataset, which is a targeted collection of data. There are a total of 70,221 images contained within this collection. The name of the second collection of data is the Repository of Molecular Brain Neoplasia Data (REMBRANDT) [36]. This set of data has MRI images of gliomas with grades II, III, and IV from 133 patients, and it has 109,021 images in total.

**Fig. 1:**
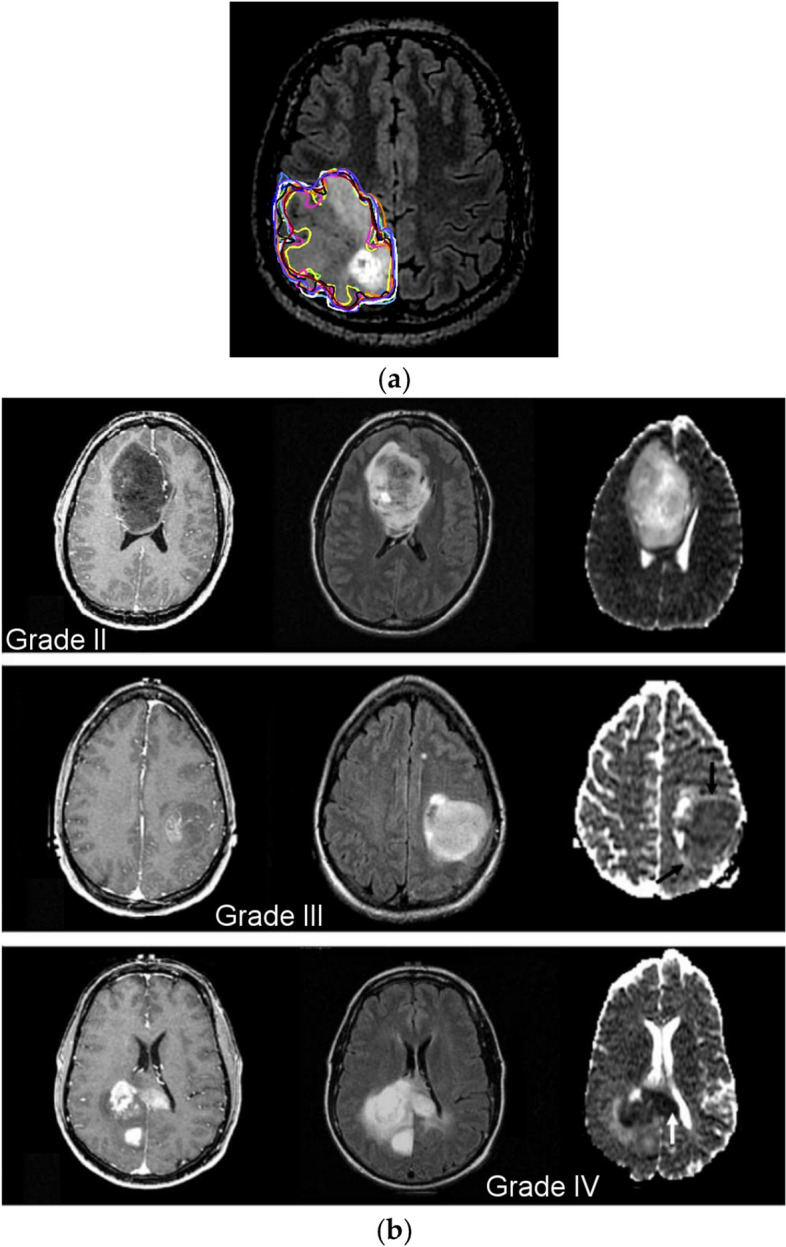
**a** Manual tumor segmentation; **b** WHO grade II (first row), grade III (second row), and grade IV (third row) brain tumors

The Cancer Genome Atlas Low-Grade Glioma dataset is the third dataset that was analyzed (TCGA-LGG) [37], and it has 242,185 MRI images of patients with low-grade gliomas (G-I and G-II) and incorporates data from 198 patients. These three datasets are part of the Cancer Imaging Archive (TCIA) project [38]. In each instance, multimodal imaging was performed, including T1-contrast-enhanced and FLAIR images [39]. The last collection of data used in this investigation consists of 3067 T1-weighted, contrast-improved images from 243 patients with three different types of brain tumors: gliomas (1427 slices), meningiomas (709 slices), and pituitary tumors (931 slices). Figure 1 depicts the different grades of brain tumors from the dataset. Totally, 3165 images are collected for the Classification-1 mode, 1743 of which are malignant tumors and 1422 of which are not. For the Classification-2 mode, 4195 images are collected. There are 910 normal images, 985 glioma images, 750 meningioma images, 750 pituitary images, and 800 metastatic images. For the Classification-3 mode, we obtain a total of 4720 images: 1712 G-II, 1296 G-III, and 1712 G-IV. Table 1 represents the dataset split-up details for the proposed model.

**Table 1:** Number of MRI images in the dataset

Dataset Split-Up			
**Classification**		**No. of Images in the Group**	**Total No. of Images**
**Mode**	**Group**		
I	Malignant	1743	3165
	Non-malignant	1422	
II	Benign	910	4195
	Glioma	985	
	Meningioma	750	
	Pituitary	750	
	Metastatic	800	
III	G-II	1712	4720
	G-III	1296	
	G-IV	1712	

### Methods

#### Convolutional neural network

**Fig. 2:**
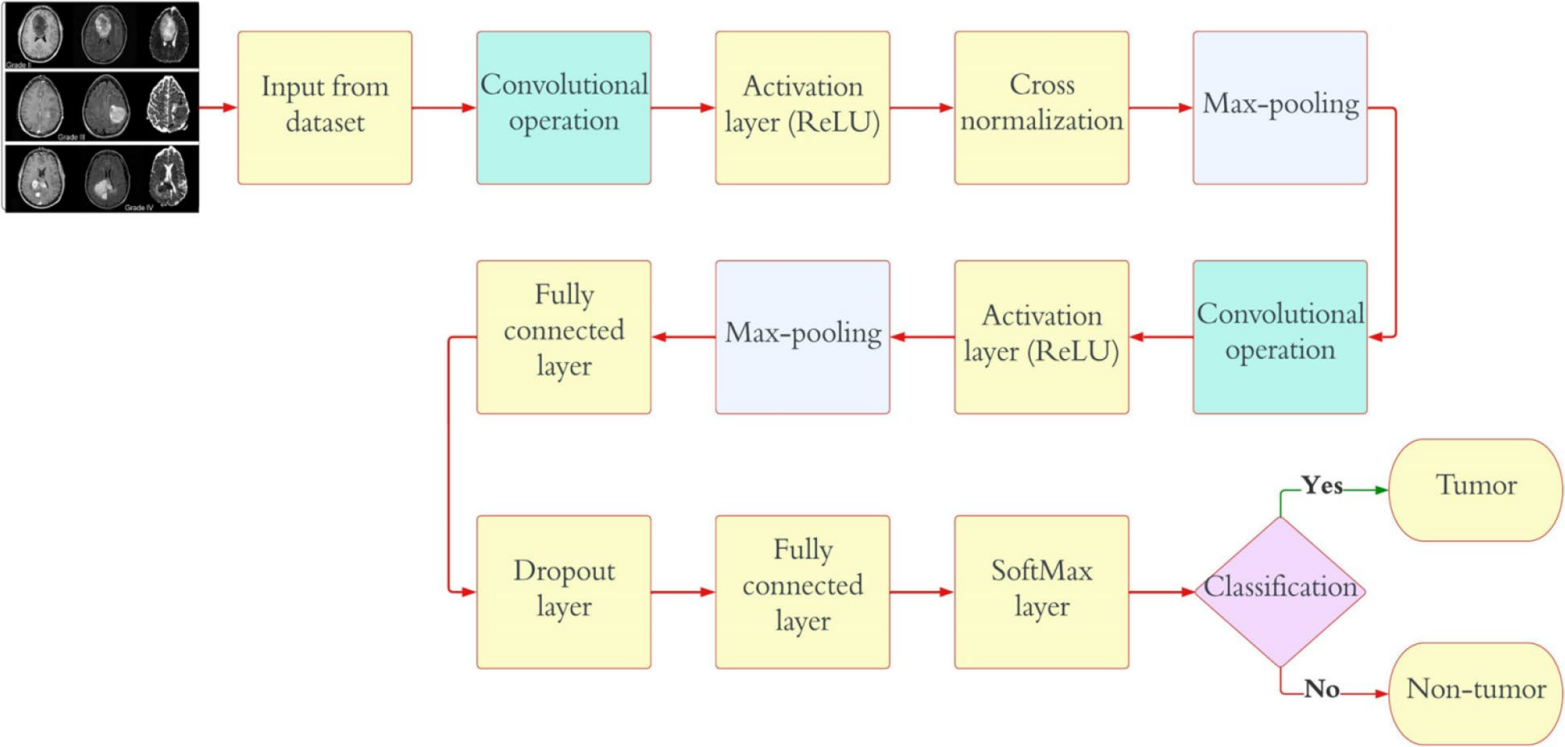
Proposed CNN model architecture for “C-1” mode

The CNN is the neural network DL model that is most frequently employed. A common CNN model has two components: classification and feature extraction. A CNN architecture has five key layers: the input layer, convolution layer, pooling layer, fully connected layer, and classification layer. The CNN provides the extraction and classification of features using successively arranged trainable layers. Convolutional and pooling layers are typically included in the feature extraction phase of a CNN, whereas fully connected and classification layers are typically included in the classification part. This proposed study suggests creating three fully automatic CNN models for classifying different types of brain tumors using MRI images. Grid search optimization tunes the key hyperparameters of the CNN models automatically. The primary of these CNN models determines whether a particular MRI image of a patient has a tumor or not, as it is employed to diagnose brain tumors. Throughout this study, this mode will be referred to as “Classification 1” (C-1). According to Fig. 2, the proposed CNN model for C-1 consists of thirteen weighted layers: one input layer, two convolution layers, two ReLU layers, one normalization layer, two max-pooling layers, two fully connected layers, one dropout layer, one softmax layer, and one classification layer.

**Fig. 3:**
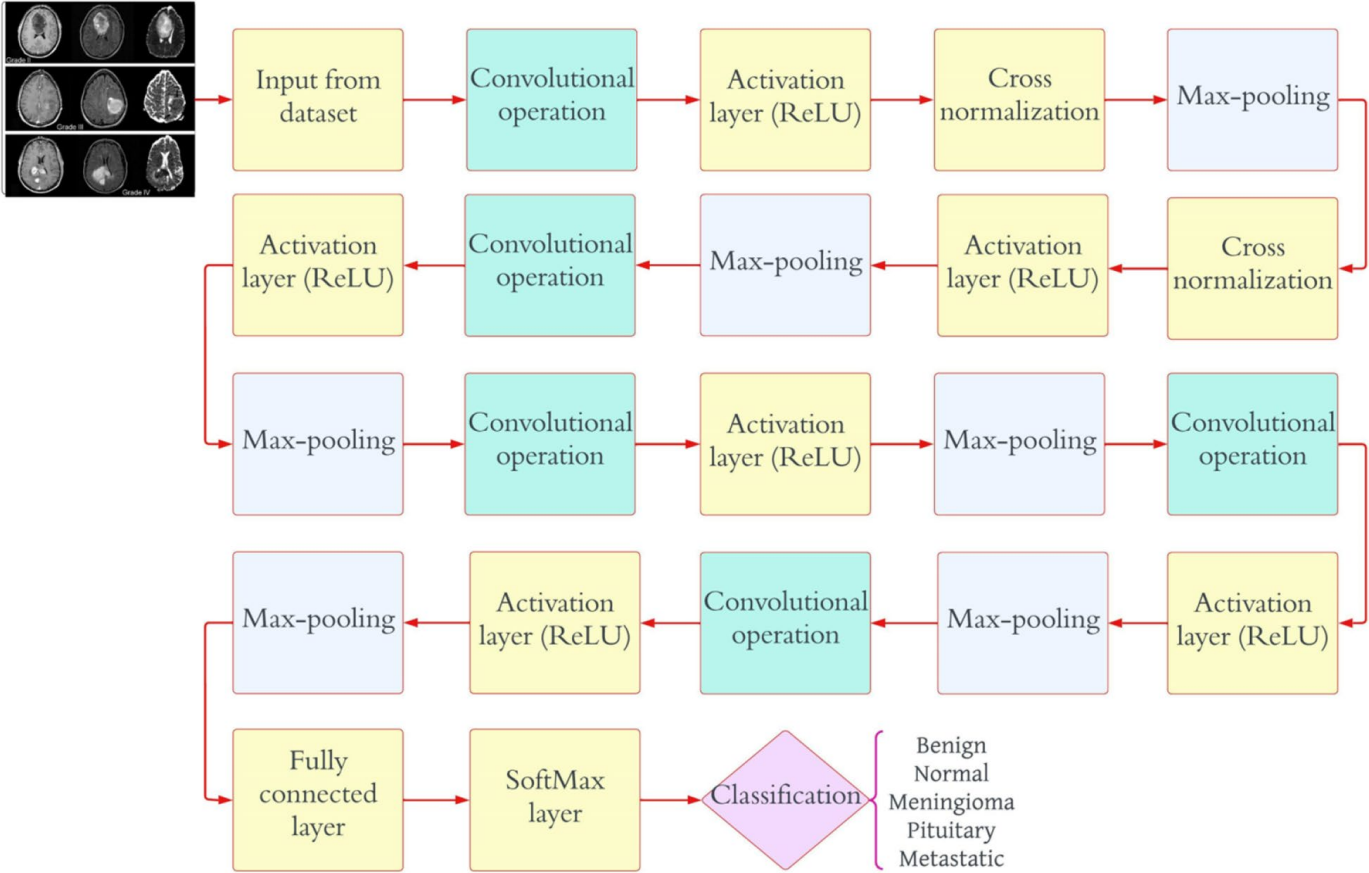
Proposed CNN model architecture for “C-2” mode

**Fig. 4:**
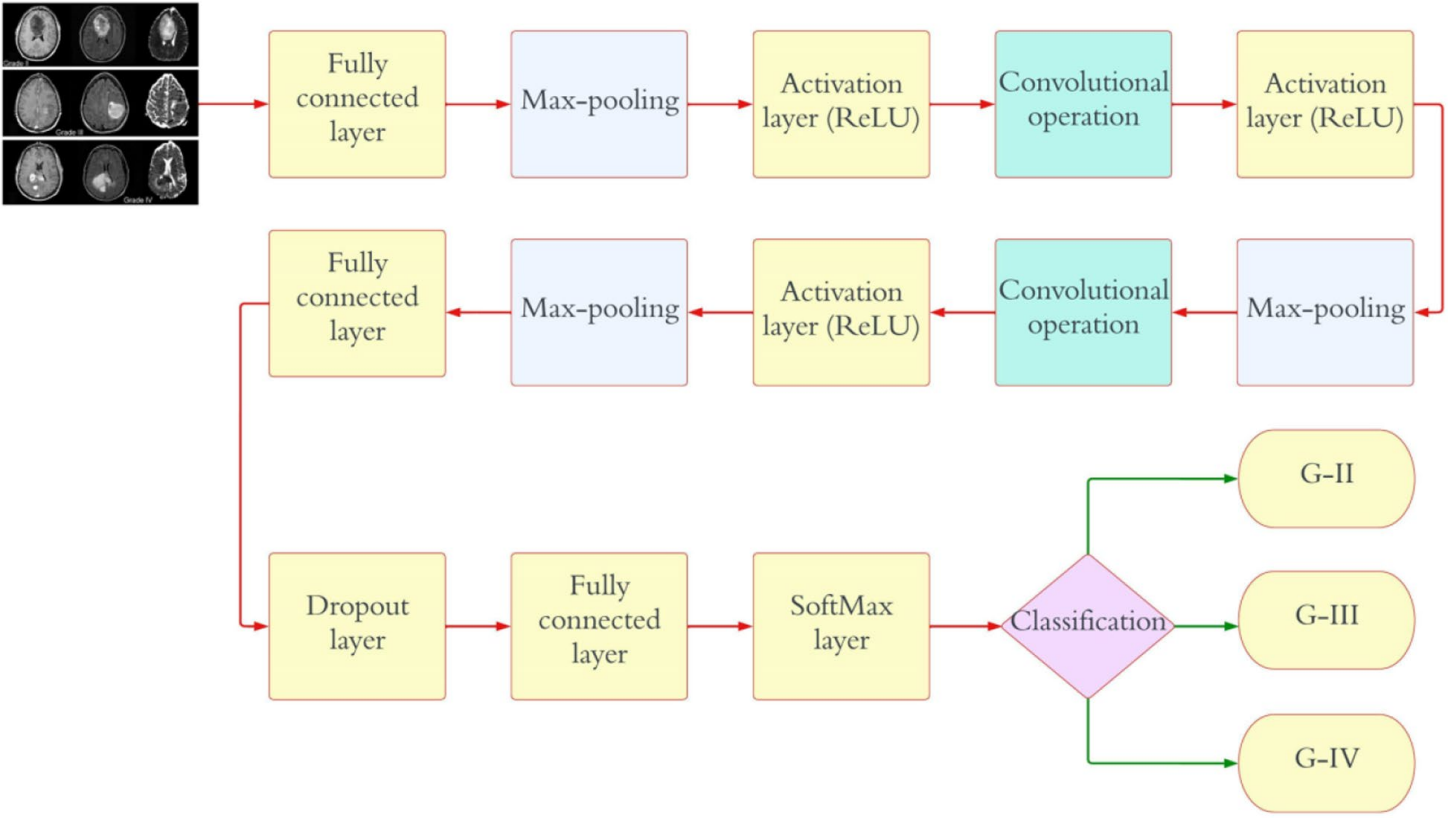
Proposed CNN model architecture for “C-3” mode

The initial CNN model is meant to classify an image into two groups, and it has two neurons in the output layer. Finally, a softmax classifier is fed the output of the fully connected layer (a two-dimensional feature vector) to determine whether a tumor is present or not. Table 2 illustrates detailed information on the CNN model. There are five distinct forms of brain tumors that are distinguished by the second CNN model: benign, malignant, meningioma, pituitary, and metastatic. Throughout this study, this mode will be referred to as “Classification 2” (C-2). As shown in Fig. 3, the proposed CNN model for C-2 contains a total of 25 weighted layers: 1 input layer, 6 convolution layers, 6 ReLU layers, 1 normalization layer, 6 max-pooling layers, 2 fully connected layers, 1 dropout layer, 1 softmax layer, and 1 classification layer. The output layer of the second CNN model has five neurons as a result of the model’s intention to classify each given image into five distinct categories. The final prediction of the tumor type is made using a softmax classifier, which receives as input the five-dimensional feature vector generated by the final fully connected layer. Table 3 illustrates detailed information on the CNN model. The third proposed CNN framework divides glioma brain tumors into three grades, which are called G-II, G-III, and G-IV. Throughout this study, this mode will be referred to as “Classification 3” (C-3). As can be seen in Fig. 4, the proposed CNN model for C-3 consists of a total of sixteen weighted layers: one input layer, three convolution layers, three ReLU layers, one normalization layer, three max-pooling layers, two fully connected layers, one dropout layer, one softmax layer, and one classification layer. The most recent CNN model has three neurons in the output layer because it is meant to divide every image into three groups. The final fully connected layer, which is a three-dimensional feature vector, is sent to the softmax classifier as an input. The softmax classifier then makes a final prediction about the tumor grade. Table 4 illustrates detailed information on the CNN model.

**Table 2:** Detailed information on CNN model employed for “C-1” mode

Layer Name	CNN Layer	Activations	Parameters (Trainable)	Total No. of Trainable Parameters
Input	227 × 227 × 3	227 × 227 × 3	nil	0
Convolutional	128 (6 × 6 × 3), stride of (4,4), with (0 0 0 0) padding	56 × 56 × 128	6 × (6 × 3) × 128 weights, 1 × 1 × 128 bias	13,954
Activation layer	Activation layer-1	56 × 56 × 128	nil	0
Normalization	Normalization (cross-channel)	56 × 56 × 128	nil	0
Max_pooling	(2 × 2) with stride of (2,2), and (0 0 0 0) padding	28 × 28 × 128	nil	0
Convolutional	96 (6 × 6 × 128), stride of (1,1), and (2 2 2 2) padding	31 × 31 × 96	2 × (2 × 128) × 96 weights, 1 × 1 × 96 bias	49,246
Activation layer	Activation layer-2	31 × 31 × 96	nil	0
Max_pooling	(2 × 2) with stride of (2,2), and (0 0 0 0) padding	15 × 15 × 96	nil	0
Fully_connected	512 Fully_connected	1 × 1 × 512	512 × 21,700 weights, 512 × 1 bias	11,060,714
Dropout	30%	1 × 1 × 512	nil	0
Fully_connected	2 Fully_connected	1 × 1 × 2	512 × 2 weights, 2 × 1 bias	1026
Softmax	Softmax	1 × 1 × 2	nil	0
Classification	Tumor or non-tumor	nil	nil	0

**Table 3:** Detailed information on CNN model employed for “C-2” mode

Layer Name	CNN Layer	Activations	Parameters (Trainable)	Total No. of Trainable Parameters
Input	227 × 227 × 3	227 × 227 × 3	nil	0
Convolutional	128 (6 × 6 × 3), stride of (4,4), with (0 0 0 0) padding	56 × 56 × 128	6 × (6 × 3) × 128 weights, 1 × 1 × 128 bias	13,952
Activation layer	Activation layer-1	56 × 56 × 128	nil	0
Normalization	Normalization (cross-channel)	56 × 56 × 128	nil	0
Max_pooling	(2 × 2) with stride of (2,2), and (0 0 0 0) padding	28 × 28 × 128	nil	0
Convolutional	96 (6 × 6 × 128), stride of (1,1), and (2 2 2 2) padding	27 × 27 × 96	6 × (6 × 128) × 96 weights, 1 × 1 × 96 bias	442,464
Activation layer	Activation layer-2	27 × 27 × 96	nil	0
Max_pooling	(2 × 2) with stride of (2,2), and (0 0 0 0) padding	13 × 13 × 96	nil	0
Convolutional	96 (2 × 2 × 96), stride of (1,1), and (2 2 2 2) padding	16 × 16 × 96	2 × (2 × 96) × 96 weights, 1 × 1 × 96 bias	36,960
Activation layer	Activation layer-3	16 × 16 × 96	nil	0
Max_pooling	(2 × 2) with stride of (2,2), and (0 0 0 0) padding	8 × 8 × 96	nil	0
Convolutional	24 (6 × 6 × 96), stride of (1,1), and (2 2 2 2) padding	7 × 7 × 24	6 × (6 × 96) × 24 weights, 1 × 1 × 24 bias	82,968
Activation layer	Activation layer-4	7 × 7 × 24	nil	0
Max_pooling	(2 × 2) with stride of (2,2), and (0 0 0 0) padding	3 × 3 × 24	nil	0
Convolutional	24 (6 × 6 × 24), stride of (1,1), and (2 2 2 2) padding	2 × 2 × 24	6 × (6 × 24) × 24 weights, 1 × 1 × 24 bias	20,760
Activation layer	Activation layer-5	2 × 2 × 24	nil	0
Max_pooling	(2 × 2) with stride of (2,2), and (0 0 0 0) padding	1 × 1 × 24	nil	0
Convolutional	32 (4 × 4 × 4), stride of (1,1), and (2 2 2 2) padding	2 × 2 × 32	4 × (4 × 24) × 32 weights, 1 × 1 × 24 bias	12,320
Activation layer	Activation layer-6	2 × 2 × 32	nil	0
Max_pooling	(2 × 2) with stride of (2,2), and (0 0 0 0) padding	1 × 1 × 32	nil	0
Fully_connected	512 Fully_connected	1 × 1 × 512	512 × 32 weights, 512 × 1 bias	16,896
Dropout	30%	1 × 1 × 512	nil	0
Fully_connected	5 Fully_connected	1 × 1 × 5	512 × 5 weights, 5 × 1 bias	2565
Softmax	Softmax	1 × 1 × 5	nil	0
Classification	Benign, glioma, pituitary, metastatic, and meningioma	nil	nil	0

**Table 4:** Detailed information on CNN model employed for “C-3” mode

Layer Name	CNN Layer	Activations	Parameters (Trainable)	Total No. of Trainable Parameters
Input	227 × 227 × 3	227 × 227 × 3	nil	0
Convolutional	128 (6 × 6 × 3), stride of (4,4), with (0 0 0 0) padding	56 × 56 × 128	6 × (6 × 3) × 128 weights, 1 × 1 × 128 bias	13,952
Activation layer	Activation layer-1	56 × 56 × 128	nil	0
Normalization	Normalization (cross-channel)	56 × 56 × 128	nil	0
Max_pooling	(2 × 2) with stride of (2,2), and (0 0 0 0) padding	28 × 28 × 128	nil	0
Convolutional	96 (6 × 6 × 128), stride of (1,1), and (2 2 2 2) padding	27 × 27 × 96	6 × (6 × 128) × 96 weights, 1 × 1 × 96 bias	46,752
Activation layer	Activation layer-2	27 × 27 × 96	nil	0
Max_pooling	(2 × 2) with stride of (2,2), and (0 0 0 0) padding	13 × 13 × 96	nil	0
Convolutional	96 (2 × 2 × 96), stride of (1,1), and (2 2 2 2) padding	16 × 16 × 96	2 × (2 × 96) × 96 weights, 1 × 1 × 96 bias	36,864
Activation layer	Activation layer-3	8 × 8 × 96	nil	0
Max_pooling	(2 × 2) with stride of (2,2), and (0 0 0 0) padding	6 × 6 × 256	nil	0
Fully_connected	512 Fully_connected	1 × 1 × 512	512 × 6144 weights, 512 × 1 bias	3,146,240
Dropout	30%	1 × 1 × 512	nil	0
Fully_connected	3 Fully_connected	1 × 1 × 3	512 × 3 weights, 3 × 1 bias	1539
Softmax	Softmax	1 × 1 × 2	nil	0
Classification	G-II, G-III, G-IV	nil	nil	0

#### Performance metric evaluation

It is essential to analyze the classification performance in image classification research to provide a rational foundation for the outcomes of the investigation. Many different performance evaluation metrics have been used for an extended period in studies involving image classification and that have evolved into standard performance evaluation metrics in studies that are similar to the prior. The proposed model used different parametric methods for evaluation, such as precision, sensitivity, and accuracy. These measures, which are generally acknowledged as standard performance evaluation metrics in image classification research, are also employed in this article in order to measure the accuracy and reliability of the classification process. Furthermore, the receiver operation characteristic (ROC) curve area, also known as the AUC of the ROC curve, is used to evaluate the models’ performance. The following are the equations containing the corresponding formulas for each of these measurements:

1 $$Accuracy=\frac{\varnothing +\beta }{\varnothing +\beta +\alpha +\gamma }$$

2 $$Specificity=\frac{\beta }{\beta +\alpha }$$

3 $$Precision=\frac{\varnothing }{\varnothing +\alpha }$$

4 $$Sensitivity=\frac{\varnothing }{\varnothing +\gamma }$$

where *ø* is true positive, *β* is true negative, *α* is false positive, and *γ* is false negative.

## Experimental Study

We implemented the proposed classification model in MATLAB2021a on a computer with the specifications of 32 GB RAM and an Intel E3-1245v6 @3.70GHz CPU.

### Optimization of the Hyperparameters

There have been several developments in the field of medical image processing that have led to the increased use of CNNs, and, as a result, some challenges have arisen in their use. The designs designed to obtain more effective outcomes are deeper, and the input images are becoming higher-quality, which leads to an increase in the amount of processing resources required. Sufficient hardware and tuning the network’s hyperparameters are essential for lowering these computing costs and maximizing results. As a result, the proposed CNN models have nearly all of their essential hyperparameters automatically set using the grid search optimization technique. When the search space for possible values is small, grid search optimization is a great way to improve a CNN’s hyperparameter optimizations. The grid search can select the superior one by training the network through a wide range of possible combinations. CNN models have architectures that are quite complicated and that have a lot of hyperparameters. In most cases, these hyperparameters can be arranged into two distinct categories: architectural hyperparameters and fine-adjustment hyperparameters. Architectural hyperparameters include the following: the number of convolutional pooling layers, the number of fully connected layers, the number of filters, the filter sizes, and the activation function. The regularization, momentum, minibatch size, and learning rate are among the fine-adjustment hyperparameters. In the current analysis, the hyperparameters of the architecture are initially tuned using Algorithm 1.

**Figure:**
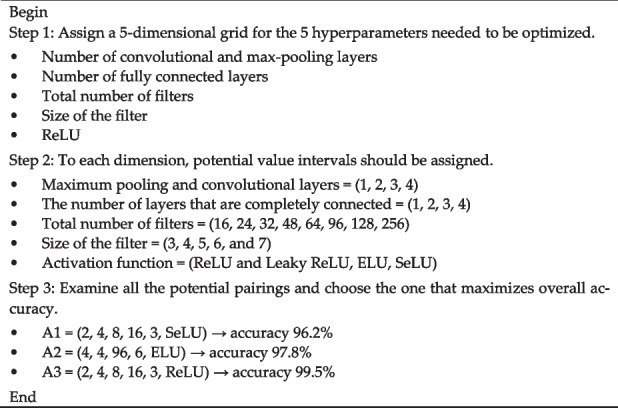
**Algorithm 1. **Architectural hyperparameters will be optimized using a grid search algorithm

After determining the architectural hyperparameters, Algorithm 2 is used to optimize the fine-adjustment hyperparameters. In this proposed study, the grid search is carried out on the training set employing a fivefold cross-validation method. The dataset is split into five different sets. Four of these sets are used for training, and the fifth set is used for testing. For the Classification-1 mode, there are 3165 images, for the Classification-2 mode, there are 4195 images, and for the Classification-3 mode, there are 4720 images. For each classification mode, the dataset is randomly split into a training set, a validation set, and a test set, with the ratio being 60:20:20. Basically, the grid search method goes through each possible setting for each parameter and finds the one that gives the best performance. In order to obtain the highest possible degree of accuracy with Algorithm 1, there are five parameters that need to be improved.

**Figure:**
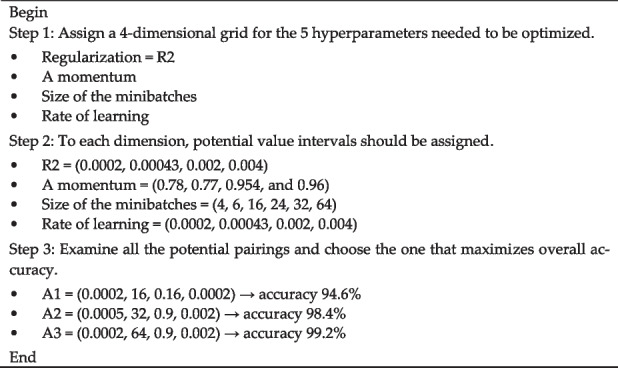
**Algorithm 2. **Architectural hyperparameters will be optimized using a grid search algorithm

Many possible combinations for these parameters, including 4, 4, 7, 5, and 4, correspondingly. As a result, the total number of possible permutations to be examined is 4 × 4 × 7 × 5 × 4, which equals 2240. Because 2240 combinations need to be checked using the fivefold cross-validation technique, the grid search algorithm created to optimize the CNN model hyper-parameters is carried out 11,200 times. Similar to the first algorithm, the second algorithm has four parameters that need to be optimized to achieve the highest level of accuracy. A wide range of permutations are possible in these parameters, for example, 4, 4, 5, and 4. As a result, the total number of possible permutations that need to be examined is 4 × 4 × 5 × 4, which equals 320. Because 320 different possible combinations need possible combinations that need to be tested using the fivefold cross-validation method, the grid search technique developed to improve the correction hyperparameters of the CNN model is carried out a total of 1600 times. As shown in Tables 5, 6 and 7, the grid search optimization algorithm found the best possible values for the hyperparameters of the C-1, C-2, and C-3 modes.

**Table 5:** The grid search-yielded optimal results for the hyperparameters for the C-1 mode

Hyperparameters	Changes in Parameter Values	Maximal Value
Layers of maximum pooling and CNN	(1, 2, 3, 4)	2
Number of layers that are completely connected	(1, 2, 3, 4)	2
Total number of filters	(8, 16, 24, 32, 48, 64, 96, 128, 256)	64, 96, 128
Intensity of filtration	(3, 4, 5, 6, 7)	6, 6
Role of activation	(ReLU, ELU, Leaky ReLU)	ReLU
Size of minibatch	(4, 6, 16, 24, 32, 64)	32
Rate of change	(0.78, 0.77, 0.95, 0.96)	0.95
Rate of learning	(0.0002, 0.00043, 0.002, 0.004)	0.0002
*R*_2_—regularization	(0.0002, 0.00043, 0.002, 0.004)	0.0002

**Table 6:** The grid search-yielded optimal results for the hyperparameters for the C-2 mode

Hyperparameters	Changes in Parameter Values	Maximal Value
Layers of maximum pooling and CNN	(1, 2, 3, 4)	6
Number of layers that are completely connected	(1, 2, 3, 4)	2
Total number of filters	(8, 16, 24, 32, 48, 64, 96, 128, 256)	16, 24, 32, 48, 64, 96, 128
Intensity of filtration	(3, 4, 5, 6, 7)	6, 6, 4, 6, 2, 6
Role of activation	(ReLU, ELU, Leaky ReLU)	ReLU
Size of minibatch	(4, 6, 16, 24, 32, 64)	64
Rate of change	(0.78, 0.77, 0.95, 0.96)	0.95
Rate of learning	(0.0002, 0.00043, 0.002, 0.004)	0.0002
*R*_2_—regularization	(0.0002, 0.00043, 0.002, 0.004)	0.002

**Table 7:** The grid search-yielded optimal results for the hyperparameters for the C-3 mode

Hyperparameters	Changes in Parameter Values	Maximal Value
Layers of maximum pooling and CNN	(1, 2, 3, 4)	3
Number of layers that are completely connected	(1, 2, 3, 4)	2
Total number of filters	(8, 16, 24, 32, 48, 64, 96, 128, 256)	64, 96, 128
Intensity of filtration	(3, 4, 5, 6, 7)	6, 6, 4
Role of activation	(ReLU, ELU, Leaky ReLU)	ReLU
Size of minibatch	(4, 6, 16, 24, 32, 64)	32
Rate of change	(0.78, 0.77, 0.95, 0.96)	0.95
Rate of learning	(0.0002, 0.00043, 0.002, 0.004)	0.004
*R*_2_—regularization	(0.0002, 0.00043, 0.002, 0.004)	0.002

### Optimized Convolutional Neural Network Outcomes

The fivefold cross-validation approach for the C-1 mode is utilized to conduct the proposed model's performance analysis. The dataset is partitioned into five different sets, four of which are utilized for training purposes, while the fifth set is placed to use for testing purposes. There are five total iterations of the experiments, and the classification performance of the mode is evaluated for each fold, and then the overall model’s average classification performance is computed. High accuracy results from the training and validation phases are meaningless if the trained and hyperparameter-tuned CNN is not tested on its ability to predict samples that have not yet been seen. Hence, to assess the effectiveness of the trained CNN to assess the trained CNN's effectiveness on predicting samples, a test dataset is randomly allocated and segregated alongside the training and validation datasets. If this step is skipped, the high accuracy may result from biased dataset assignment. Table 8 displays the results of randomly splitting the 3165 images from the study into the training, validation, and test sets in the ratio of 60:20:20 for the C-1 mode.

**Table 8:** Training, validating, and testing phases of proposed CNN model

Dataset Split-Up				Training, Validation, and Testing Modes		
**Classification**		**No. of Images in the Group**	**Total No. of Images**	**Training Mode (60%)**	**Validation Mode (20%)**	**Test Mode (20%)**
**Task**	**Group**					
I	Malignant	1743	3165	1899	633	633
	Non-malignant	1422				
II	Benign	910	4195	2517	839	839
	Glioma	985				
	Meningioma	750				
	Pituitary	750				
	Metastatic	800				
III	G-II	1712	4720	2832	944	944
	G-III	1296				
	G-IV	1712				

**Fig. 5:**
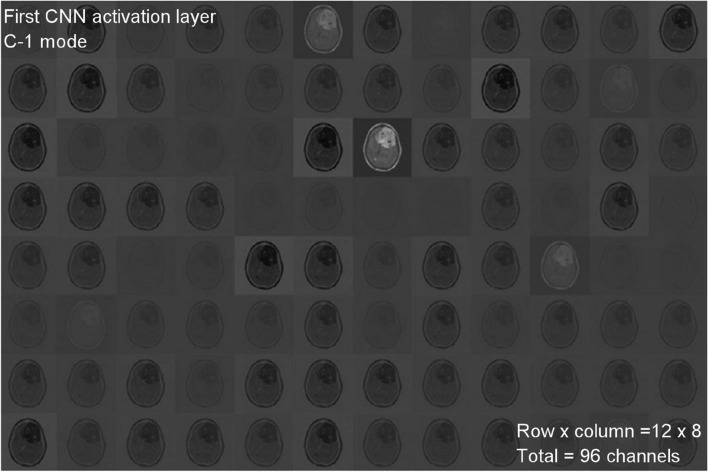
First CNN activation layer for C-1 mode

**Fig. 6:**
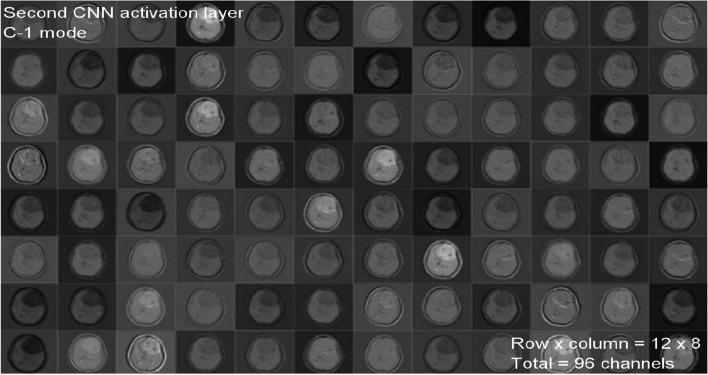
Second CNN activation layer for C-1 mode

A total of 299 images are taken randomly from the dataset for each category, and then those images are used for testing. The activations of the CNN’s convolution layers can be displayed for a better view of the features that the CNN has learned due to its training. With this representation, the researcher may easily observe the network’s progress. Figures 5 and 6 each depict the activations of the first and second convolutional layers. One of the images in the grid serves as a representation of the channel’s outcome. White areas represent highly positive activations, while grey areas represent moderately activated channels. While the first convolutional layer of the CNN is used to learn features such as color and edges, the second convolutional layer is used to learn more complex information, such as the borders of brain tumors. The succeeding (deeper) convolutional layers build up their features by merging the features learned by the earlier convolutional layers.

**Fig. 7:**
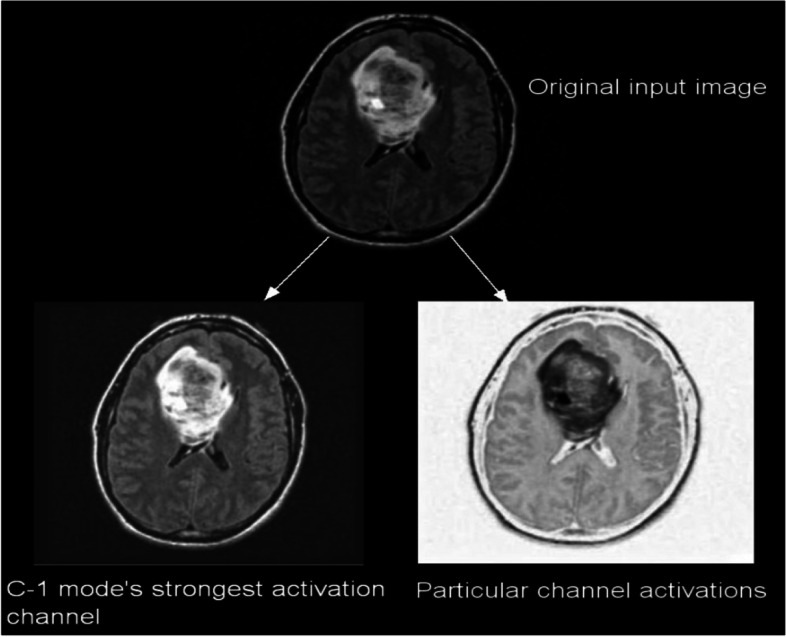
C-1-mode strongest and moderate images from original input image

Figure 5 shows 96 of the 128 channels in the CNN’s first convolutional layer running in C-1 mode. This layer contains a total of 128 channels. Figure 6 shows an image of the second convolutional layer of the network, which has 96 channels. Every layer of the CNN is composed of channels, which are arrays in two dimensions. One of the images in Fig. 5 represents the output of each channel in the first convolutional layer. In these images, strong positive activations are shown by white pixels, and strong negative activations are shown by black pixels. Similarly, grey pixels on the input image indicate channels that are not highly active. Figure 7 depicts the activations of a particular channel and the channel with the most significant activation in the first convolutional layer. The presence of white pixels in the channel of Fig. 7 demonstrates that this channel is highly activated at the tumor's location. Although the CNN was never instructed to learn about tumors, it is possible to conclude that it has picked up on the fact that tumors have distinguishing qualities that allow it to differentiate between different categories of images.

**Fig. 8:**
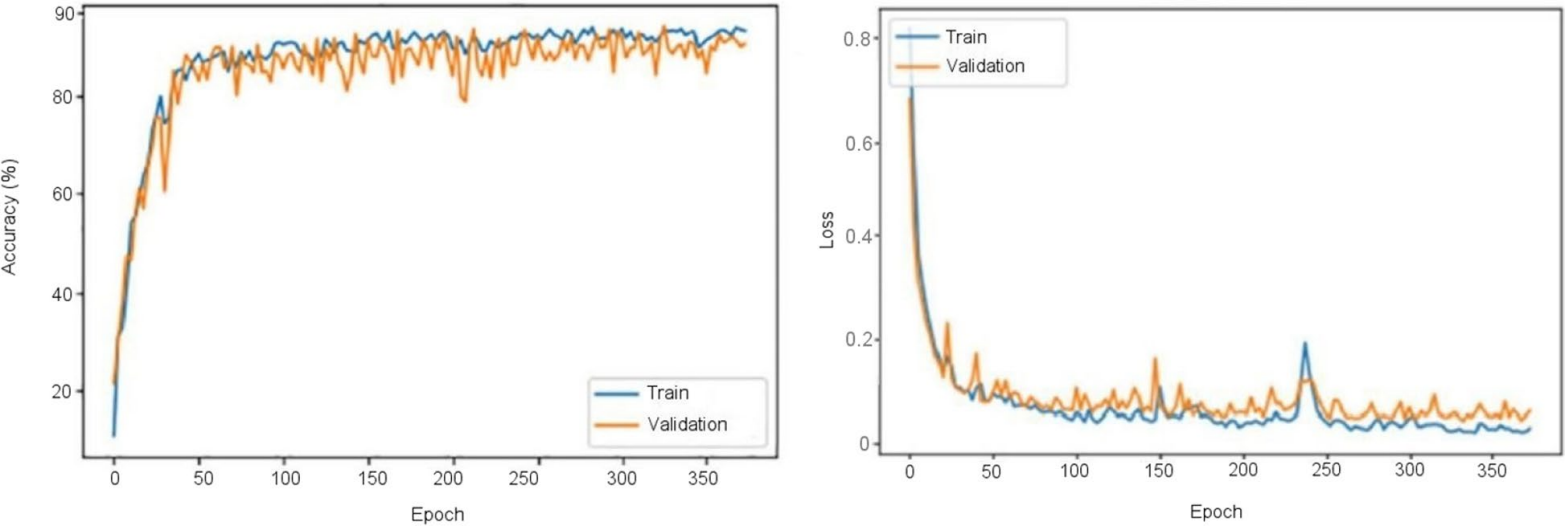
C-1-mode accuracy and loss curves

**Fig. 9:**
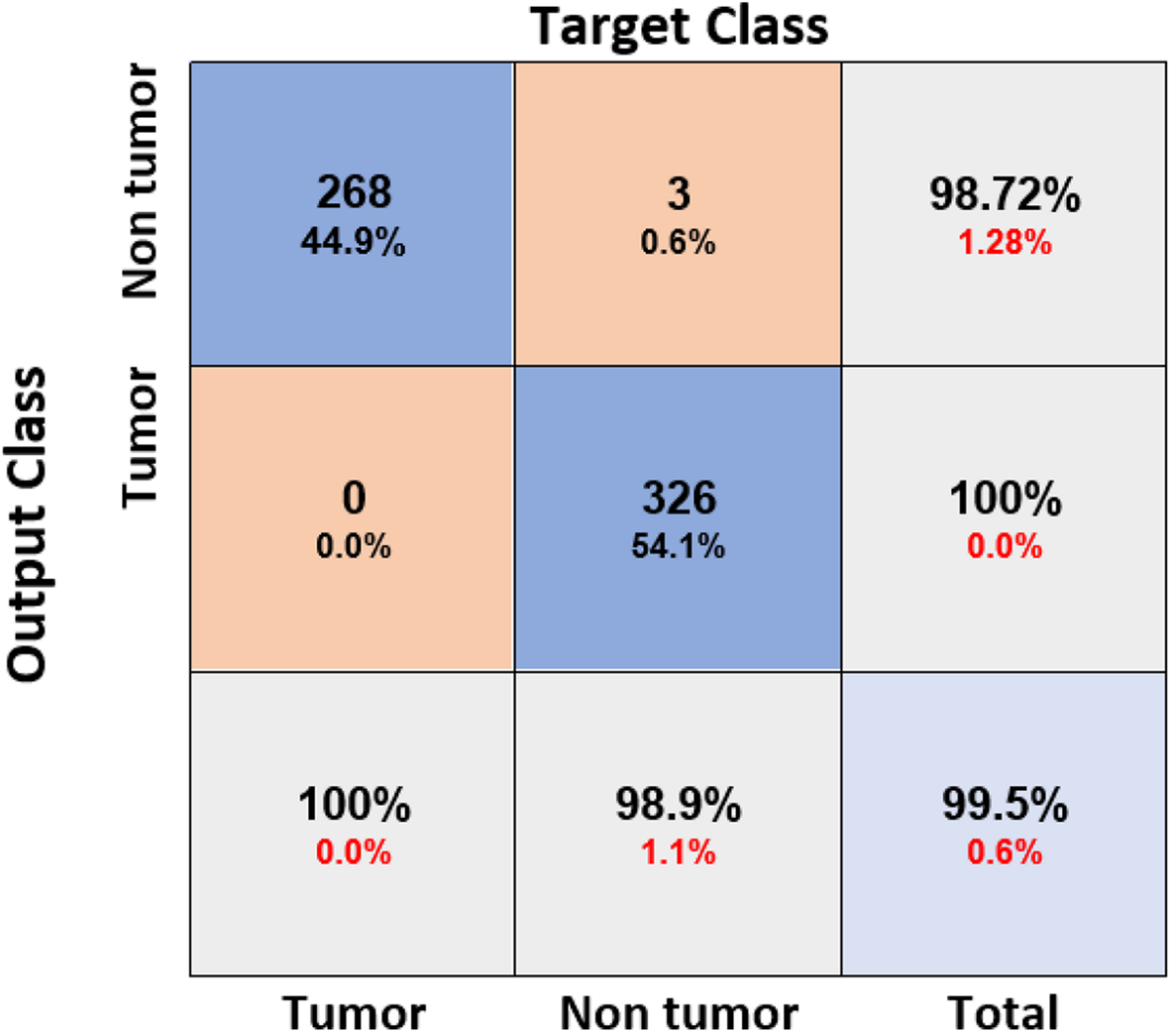
C-1 confusion matrix

**Fig. 10:**
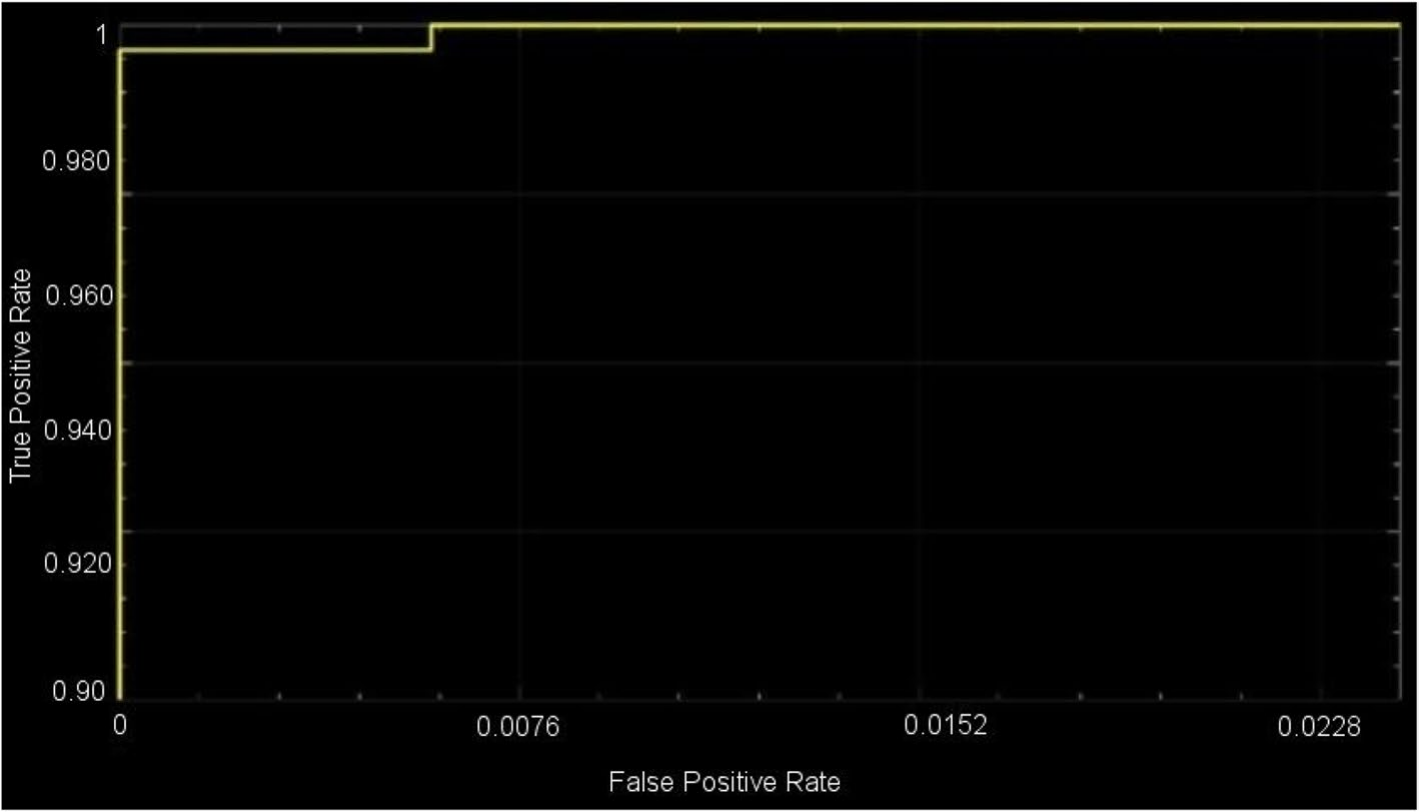
C-1-mode average of ROC curve

These CNNs are able to discover helpful characteristics on their own, unlike earlier artificial neural network methods that typically required manual design to fit a particular mode. In this proposed article, learning to recognize tumors improves the ability to distinguish between a tumor image and non-tumor image. After the process of classification has been completed, the efficiency of the CNN models must be evaluated using different reliable approaches. The metrics, like the specificity, sensitivity, precision, and accuracy measures, as well as the area under the ROC curve, are used to perform the performance evaluation of the proposed model. The proposed CNN’s loss and accuracy plots for the C-1 mode are shown in Fig. 8. After 340 iterations, the model proposed for C-1 was able to classify with a 99.53% accuracy. It is pretty clear, as shown in Fig. 8, that approximately 250 iterations are required to reach an almost perfect level of accuracy. Figure 9 depicts the confusion matrix for the Classification-1 mode. As can be seen in Fig. 10, the area under the ROC curve has a value of 0.9995 for its AUC. The results presented here demonstrate that the recommended CNN model is capable of identifying brain tumors. Table 9 shows the measures of the accuracy, such as the true positive (TP), true negative (TN), false positive (FP), false negative (FN), accuracy (Acc), specificity (Sp), sensitivity (Se), and precision (Pr). Figure 10 depicts the ROC curve for the Classification-1 (C-1) task.

**Table 9:** Proposed CNN model parameter metric outcomes for all classification modes

Metrics	Classes	TP	TN	FP	FN	Acc	Sp	Se	Pr	Total
I	Malignant	268	326	3	0	99.50	99.09	100.00	98.89	268
	Non-malignant	326	268	0	3	99.50	100.00	99.09	100.00	329
II	Benign	183	598	8	8	97.99	98.68	95.81	95.81	191
	Glioma	132	650	10	12	97.26	98.48	91.67	92.96	144
	Meningioma	138	643	6	14	97.50	99.08	90.79	95.83	152
	Pituitary	160	598	24	11	95.59	96.14	93.57	86.96	171
	Metastatic	127	643	11	14	96.86	98.32	90.07	92.03	141
III	G-II	332	574	9	8	98.16	98.46	97.65	97.36	340
	G-III	248	679	0	0	100.00	100.00	100.00	100.00	248
	G-IV	330	580	8	9	98.17	98.64	97.35	97.63	339

**Fig. 11:**
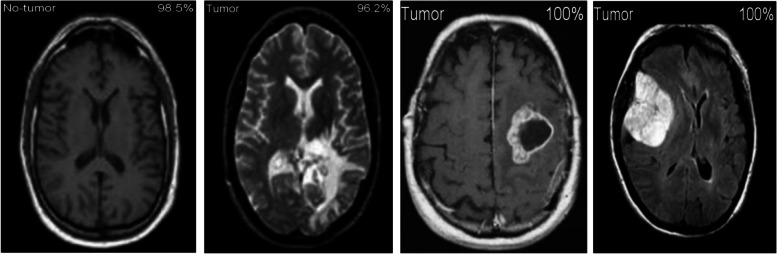
The results of classification and predictions for the probabilities of four different test images for the C-1 mode

**Fig. 12:**
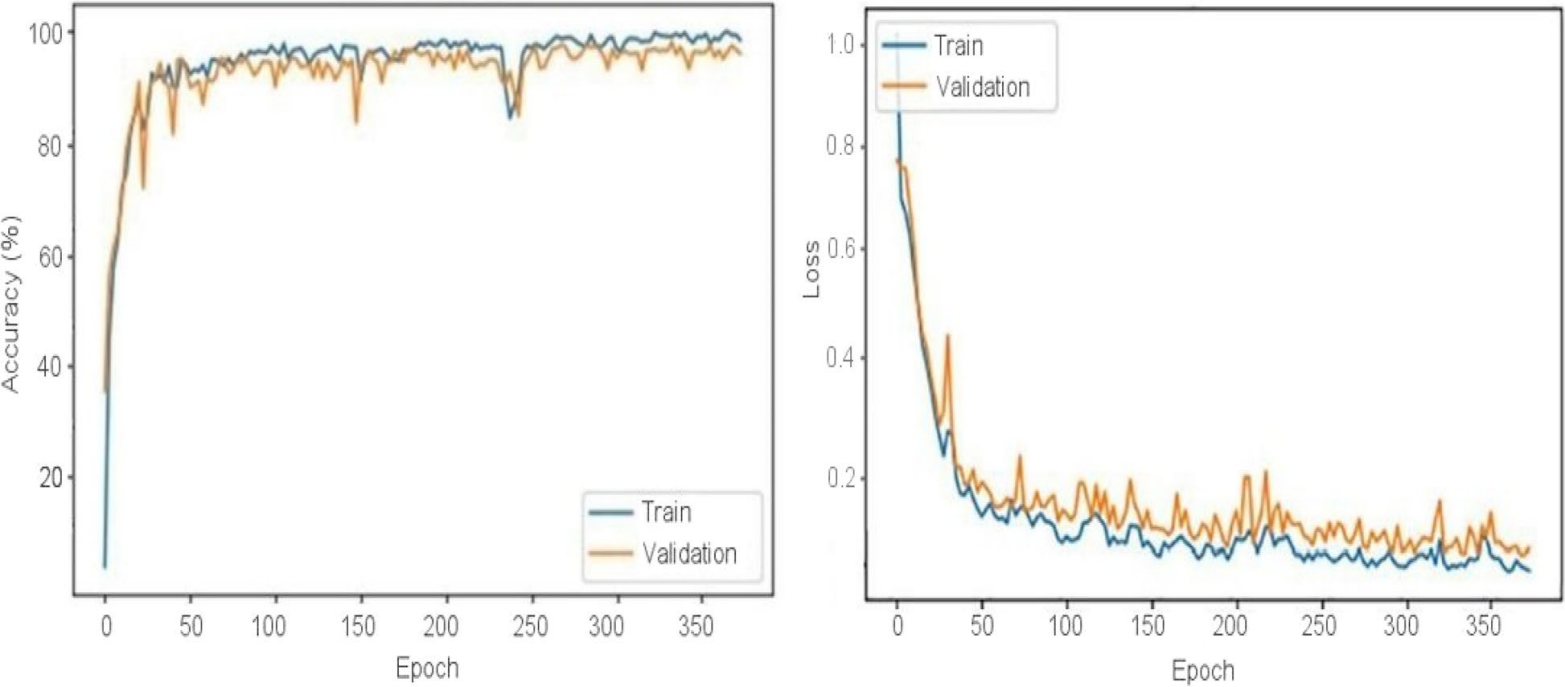
C-2-mode accuracy and loss curves

**Fig. 13:**
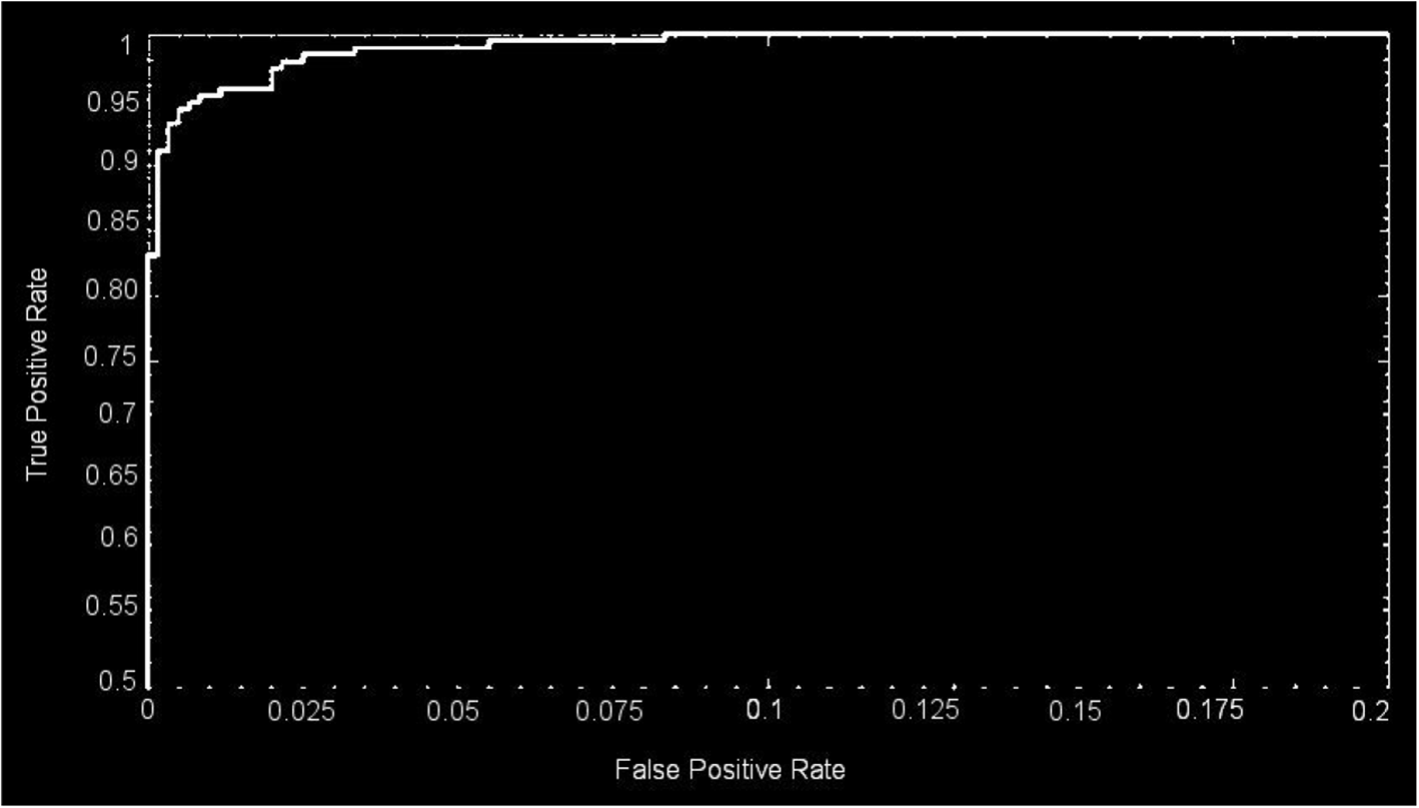
C-2-mode average of ROC curve

**Fig. 14:**
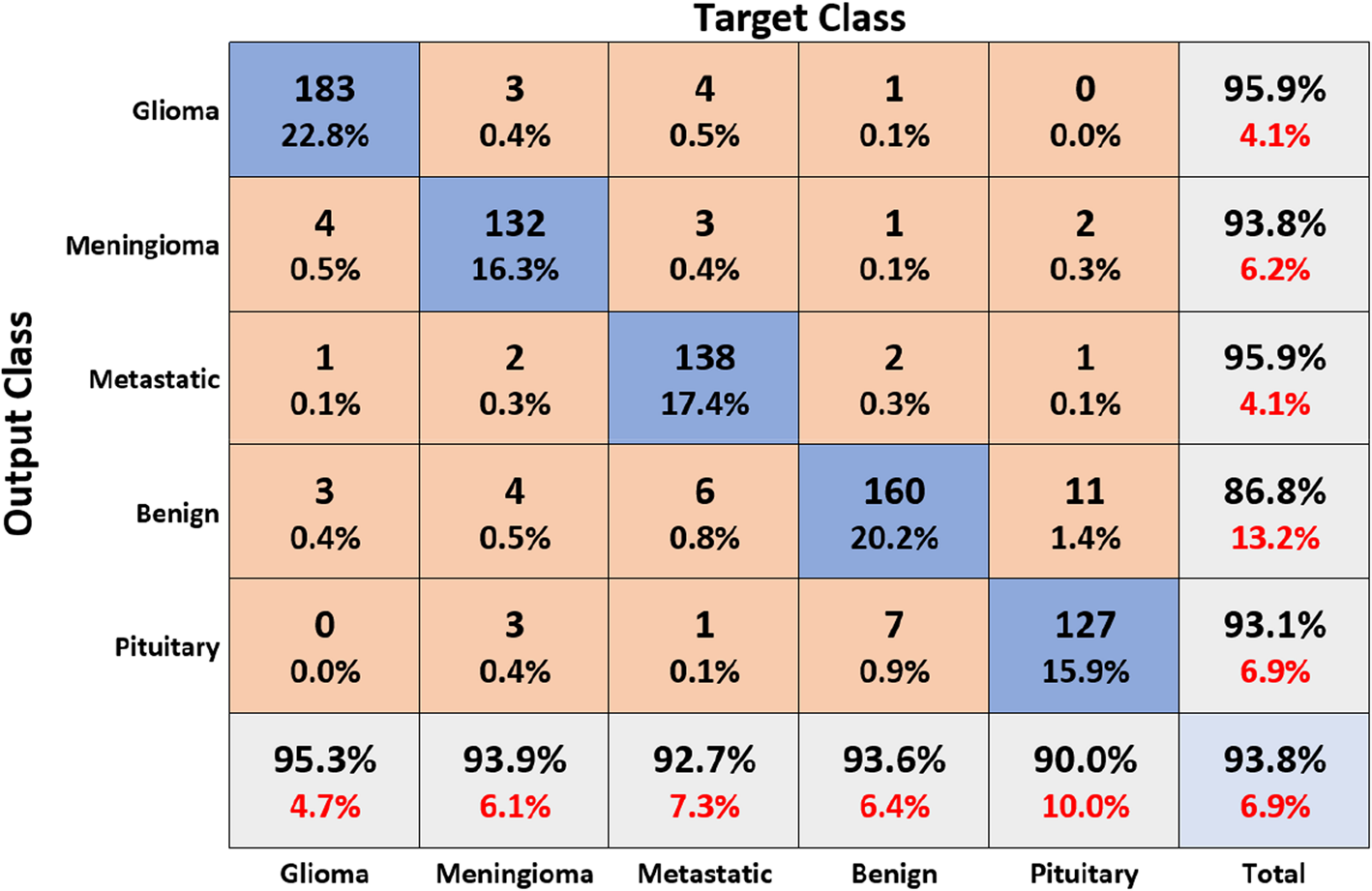
C-2 confusion matrix

Figure 11 shows the results of the classification and the predicted probabilities for each of the four tests conducted in C-1 mode. Implementing the fivefold cross-validation method for the C-2 mode evaluates the effectiveness of the proposed framework. The dataset is partitioned into five sets, four of which are utilized for training purposes, while the fifth set is placed for testing purposes. There are five total iterations of the experiments. The classification performance of the job is evaluated for each fold, and then the overall model’s average classification performance is computed. As indicated in Table 8, there are sufficient images for the C-2-mode training, validation, and test sets to be randomly divided in a ratio of 60:20:20 for a sample size of 4195. From the dataset of each class that will be used to test the model, 158 images are randomly selected to be removed. The accuracy and loss plots of the suggested CNN model for the C-2 task are displayed in Fig. 12. The proposed CNN method for the C-2 mode achieves a 93.81% accuracy in classification after 294 iterations. As shown in Fig. 13, the area under the ROC curve has a value of 0.9981. These findings demonstrate the proposed CNN model's capability to classify brain tumor types. Figure 14 depicts a confusion matrix, and Table 9 lists the many measures of precision, such as TP, TN, FP, FN, Acc, Sp, Se, and Pr. According to Table 9, an accuracy of 97.26% is attained when classifying a glioma, 97.50% when classifying a meningioma, 96.86% when classifying metastasis, 97.99% when classifying a healthy brain, and 95.59% when classifying the pituitary tumor type for the C-2 mode. Figure 14 depicts the ROC curve for the Classification-2 (C-2) task.

**Fig. 15:**
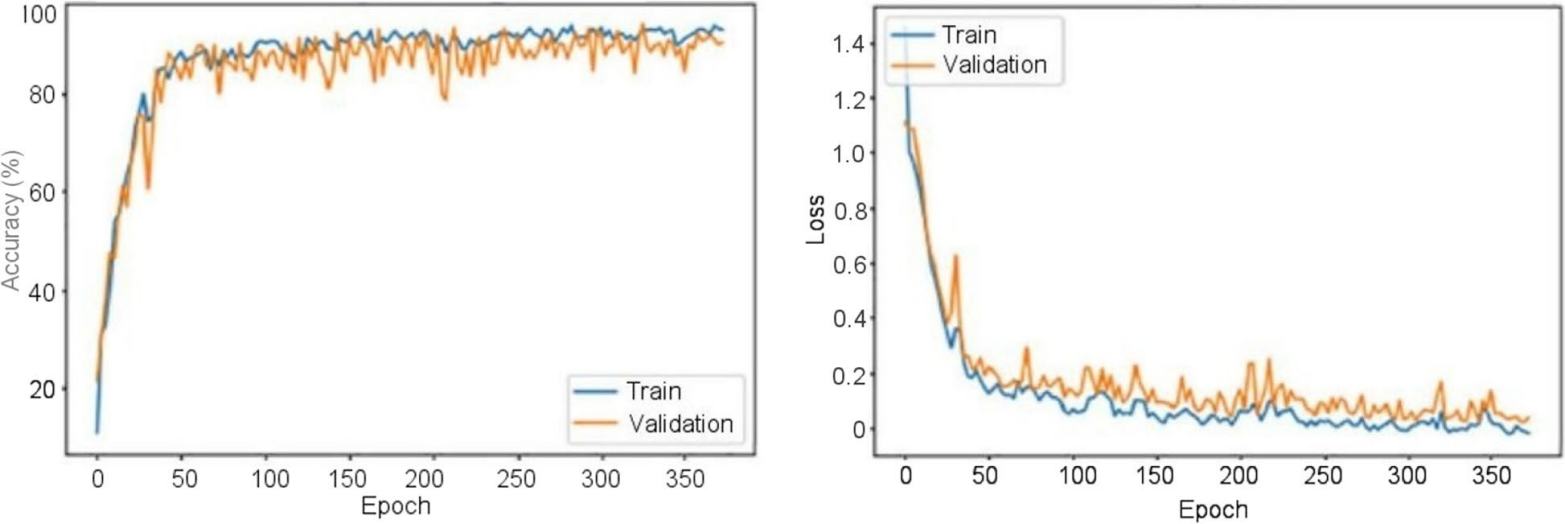
C-3-mode accuracy and loss curves

**Fig. 16:**
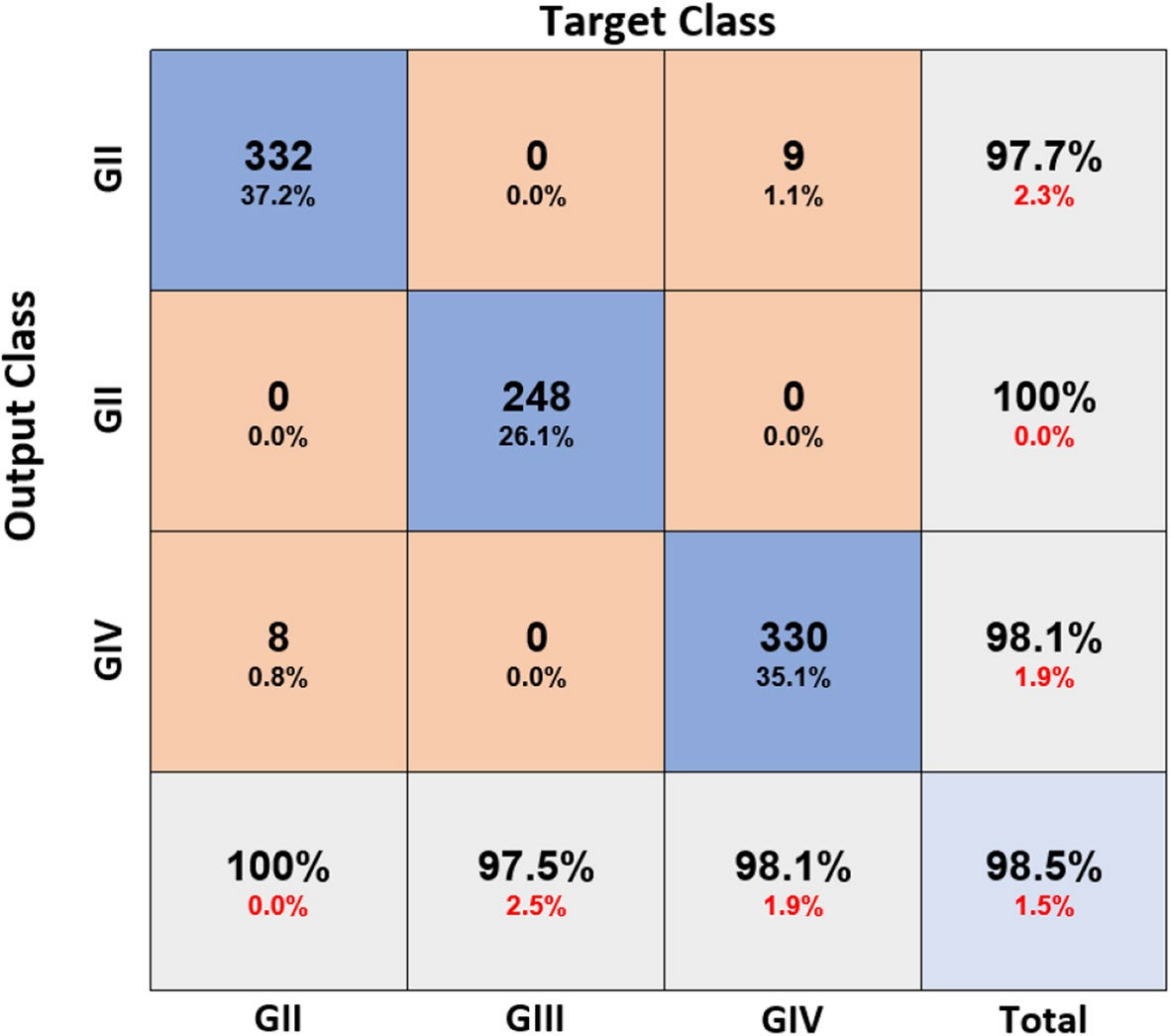
C-3 confusion matrix

**Fig. 17:**
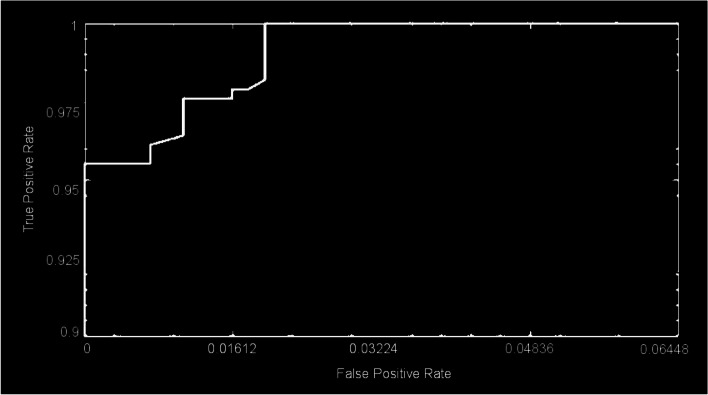
C-3-mode average of ROC curve

**Fig. 18:**
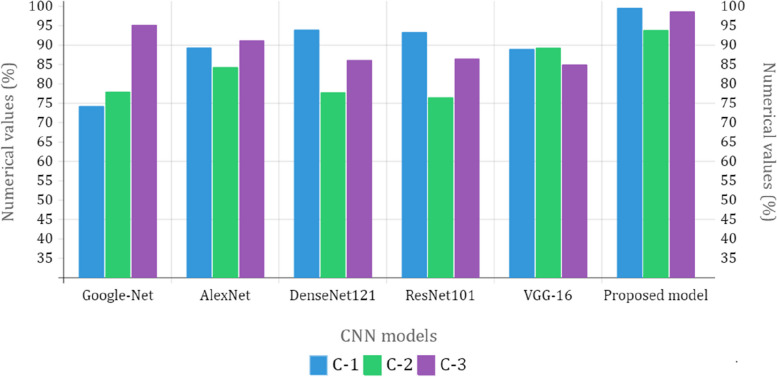
Graphical illustration of proposed and existing models’ outcome comparison

The fivefold cross-validation process for the C-3 mode is utilized to evaluate the efficacy of the proposed models. The dataset is partitioned into five different sets, out of which four are used for training and the fifth is used for testing. There are five total iterations of the experiments. Following an analysis of the classification performance of the mode for each fold, an average classification performance for the model is computed. For the C-3 mode, sufficient images can be randomly divided into training, validation, and test sets in the proportions 60:20:20, as indicated in Table 8, randomly excluding three hundred and five images from the dataset of each class to be utilized to evaluate the model. The loss and accuracy graphs of the proposed CNN for the C-3 mode are shown in Fig. 15. Figure 16 depicts the confusion matrix for the C-3 mode. The proposed approach for the C-3 mode obtains a classification accuracy of 98.16% after 344 iterations. Figure 17 depicts the ROC curve for the Classification-3 (C-3) task. Table 9 shows that an accuracy of 98.16% is reached when classifying grade II, 100% when classifying grade III, and 98.17% when classifying grade IV for brain tumor grades in the C-3 mode. The three different classification outcomes of the proposed CNN model were compared with other conventional CNN approach outcomes to evaluate the proposed system classification ability. To achieve this goal, the same experiments were performed with the same dataset, utilizing well-known and popular pretrained CNN models, such as AlexNet, DenseNet121, ResNet-101, VGG-16, and GoogleNet. Table 10 illustrates the performance metric outcome comparison of the proposed CNN model with existing CNN approaches. Figure 18 depicts the graphical representation of the proposed and existing models’ result comparison.

**Table 10:** Performance metric outcome comparison of the proposed CNN model with existing CNN approaches

CNN Models	C-1 Mode		C-2 Mode		C-3 Mode	
	**Acc (%)**	**AUC**	**Acc (%)**	**AUC**	**Acc (%)**	**AUC**
GoogleNet	74.21	0.8108	77.89	0.8212	95.12	0.9617
AlexNet	89.23	0.8989	84.24	0.8501	91.08	0.9772
DenseNet121	93.89	0.9412	77.67	0.8122	86.07	0.8809
ResNet101	93.29	0.9442	76.45	0.8115	86.42	0.881
VGG-16	88.87	0.9201	89.19	0.8112	84.87	0.8663
Proposed CNN approach	99.53	0.9994	93.81	0.9984	98.56	0.9993

The results shown in Table 10 illustrate that the proposed CNN models outperform other networks in every classification mode. The pretrained DenseNet121 model, which obtains a 93.89% classification accuracy in the brain tumor detection test (C-1 mode), is the model that is closest to the suggested model. The pretrained VGG-16 model obtains an 89.19% accuracy in the brain tumor-type classification mode (C-2 mode). It is the model that is closest to the proposed CNN model. After the proposed CNN model, the pretrained GoogleNet model achieves a classification accuracy of 95.12%, making it the best network available for grading tumors (C-3 mode). It is clear that the proposed CNN models are better than the pretrained networks, which were built and trained using generic datasets and methods for a wide range of image classification tasks. Table 11 illustrates the proposed and existing model outcome comparison. The proposed CNN models, conversely, were designed to deal with more specific issues, like identifying and defining various types and stages of brain tumors. Finally, MRI images of brain tumors are used to train and evaluate the proposed models.

**Table 11:** Comparison of the proposed model with existing studies

Author	Year	Datasets	Method	Classification of Accuracy (%)
Ahmed Wasif Reza [12]	2023	Kaggle, Figshare	VGG-16 architecture	96.70
Mahmoud Khaled Abd-Ellah [13]	2018	RIDER, REMBRANDT, and BraTS	ECOC-SVM	97.98
Takowa Rahman [27]	2023	Kaggle, REMBRANDT	CNN + 6 pretrained models	97.12
Anand Deshpande [17]	2021	RIDER and BraTS	Discrete cosine transform-based image fusion combined with CNN	98.14
Proposed model	2023	Figshare, REMBRANDT, TCGA-LGG, TCIA	Hybrid CNN	98.56

## Conclusions

In this research, we propose a multi-classification method for identifying brain tumors at an early stage using (CNN) models, in which nearly all the hyperparameters are automatically optimized via grid search. By using publicly available medical imaging datasets, three reliable CNN models have been designated to perform three distinct brain tumor classification tasks. A high level of accuracy, such as 99.53%, can be attained in the process of detecting brain tumors. In addition, a remarkable accuracy of 93.81% is achieved when classifying brain MR images into the categories of glioma, meningioma, pituitary, normal brain, and metastatic. The final step is grading glioma brain tumors, which can be performed with an accuracy of 98.56% for grades II, III, and IV. A good number of medical images are used to train and test the CNN models that are being proposed. Results from the proposed CNN models and comparisons with current methods show that CNN models made with the proposed optimization framework work well. In this work, CNN models were made that can help clinicians and radiologists check primary screenings for multiple types of brain tumors.

## Data Availability

The datasets used during the current study are available from the corresponding author upon reasonable request.
